# A Synergism between Adaptive Effects and Evolvability Drives Whole Genome Duplication to Fixation

**DOI:** 10.1371/journal.pcbi.1003547

**Published:** 2014-04-17

**Authors:** Thomas D. Cuypers, Paulien Hogeweg

**Affiliations:** Theoretical Biology and Bioinformatics Group, Utrecht University, Utrecht, the Netherlands; University of California Davis, United States of America

## Abstract

Whole genome duplication has shaped eukaryotic evolutionary history and has been associated with drastic environmental change and species radiation. While the most common fate of WGD duplicates is a return to single copy, retained duplicates have been found enriched for highly interacting genes. This pattern has been explained by a neutral process of subfunctionalization and more recently, dosage balance selection. However, much about the relationship between environmental change, WGD and adaptation remains unknown. Here, we study the duplicate retention pattern postWGD, by letting virtual cells adapt to environmental changes. The virtual cells have structured genomes that encode a regulatory network and simple metabolism. Populations are under selection for homeostasis and evolve by point mutations, small indels and WGD. After populations had initially adapted fully to fluctuating resource conditions re-adaptation to a broad range of novel environments was studied by tracking mutations in the line of descent. WGD was established in a minority (≈30%) of lineages, yet, these were significantly more successful at re-adaptation. Unexpectedly, WGD lineages conserved more seemingly redundant genes, yet had higher per gene mutation rates. While WGD duplicates of all functional classes were significantly over-retained compared to a model of neutral losses, duplicate retention was clearly biased towards highly connected TFs. Importantly, no subfunctionalization occurred in conserved pairs, strongly suggesting that dosage balance shaped retention. Meanwhile, singles diverged significantly. WGD, therefore, is a powerful mechanism to cope with environmental change, allowing conservation of a core machinery, while adapting the peripheral network to accommodate change.

## Author Summary

The evolution of eukaryotes is characterized by drastic changes in their genome content. Genome expansions have often occurred by duplication of the entire genome. It is generally not know whether organisms gain any adaptive advantage from these mutations. However, they appear to become fixed in response to environmental change. Many interesting whole genome duplications happened long ago in eukaryotic evolutionary history during periods of turbulent genome and species evolution. Genomic data analysis alone cannot resolve the evolutionary mechanisms and consequences of whole genome duplication. Here, we modeled evolution with whole genome duplications in a Virtual Cell model. Simulating populations that undergo a range of different environmental changes we found that next to often increasing fitness directly, whole genome duplications made lineages more evolvable and hence more able to adapt to harsh new environments. Although most duplicates are deleted in subsequent evolution, genes with many interaction partners were retained preferentially, increasing regulatory complexity. Interestingly however, we found that innovation happened most likely in the more loosely connected and less essential genes.

## Introduction

Eukaryotic genomes differ up to an astonishing 200000 fold in the amount of their DNA, by far the widest range within all domains of life [1]. In eukaryotic evolution large changes in genome size have heralded major transitions, starting with the radiation from a common ancestor of the eukaryotic supergroups within a short evolutionary timespan [2], [3]. Subsequent dramatic radiations of animals in the Cambrian explosion and flowering plants have also been preceded by extensive increases in genome size [4], [5]. But even within narrow taxonomic bounds remarkable levels of genome size variability exist, such as the seven fold difference within the Brachionus plicatilis species complex [6].

What are the evolutionary mechanisms underlying this flexibility in genome size and how does it affect the dynamics of eukaryotic evolutionary history? Ever since Ohno first proposed that the genome of the vertebrate ancestor had undergone two rounds (2R) of duplication [7], evidence of the pervasiveness of WGD in eukaryotic evolution has been mounting. The 2R hypothesis itself has been strongly backed by recent phylogenetic studies [8], [9]. Similarly, species radiations of angiosperms [10], teleost fish [11] and yeasts [12] have all been associated with rounds of WGD. Especially in plants, the transition to polyploidy appears to be remarkably frequent. Therefore, in addition to all flowering plants being of paleopolyploid descent [10], [13], it is estimated that up to a third of all extant plant species underwent polyploidization since their most recent speciation [14].

Recognizing the ubiquity of WGD in eukaryotic evolution, it becomes crucial to understand the mechanisms that lead to their fixation in evolving populations. Data on plants suggest that changing environmental conditions can give rise to the establishment of polyploid lineages. For example, polyploid incidence is increased in harsher and newly arisen environments such as the arctic [15] and on islands created by volcanic activity [16] or at the ecological limits of non-polyploid parent species (reviewed extensively in [17]). An extensively studied case of ancient WGD that happened in the ancestor of S. cerevisiae was shown to potentially yield a direct adaptive benefit when a novel, glucose rich environment arose [18], [19]. However, direct adaptive benefits may not play a role in other historic cases of WGD which instead may be better explained by a general increase in evolvability. This may be the reason why a burst of WGDs in plants appears to coincide with the K-T boundary event, explaining the success of these lineages in overcoming the drastic change in climate conditions [20], [21].

Most duplicates that arose from an ancient WGD event will have typically returned to a single copy state, thereby eroding the signal of WGD [12]. Remaining ohnolog (duplicates arising from WGD) fractions, ranging from 16% in yeast [22] to more than 50% in P. tetraurelia [23], have been studied to gain insights in the potential adaptive benefits of WGD and evolutionary forces that play a role in post WGD genome evolution. In general, duplicate retention post WGD is not equal for all gene classes. A pattern found across species is an over-retention of transcription factors (TFs) and signaling genes in duplicate [24], [25], [26], [27], [28], [23].

Neutral loss of subfunctions in both copies, for example losing different subset of target genes for TFs could enforce this retention [29] and has indeed been observed for Arabidopsis ohnologs [30], [31]. However, a characteristic reciprocal relationship between the retention of duplicates resulting from WGD and small scale duplication (SSD) can not be easily explained by subfunctionalization. For example, TFs have been overretained post WGD, while underretained post SSD [32], [33], [34], [28], [35], [26]. This pattern would, however, be predicted by the gene balance theory, because the two modes of duplication affect the balance between interacting gene products differently. Whereas a WGD should generally retain the balance between highly interacting genes, SSD most likely disrupts this balance by only increasing the dosage of a few genes [34], [26], [36], [28]. This suggests that dosage balance selection could drive retention of duplicates post WGD [28], [37] at least on short evolutionary timescales. Transient retention due to dosage balance selection can increase the chance that duplicates subfunctionalize or even neofunctionalize, further increasing the likelihood of duplicate retention [27].

How gene balance constraints affect gene divergence and loss remains, however, poorly understood. One important reason is that adaptive and neutral genome evolution post WGD can produce mixed conservation patterns [27]. In short, we lack a comprehensive mechanistic understanding of the causes and consequences of WGD when populations adapt to environmental change as well as its impact on long term genome evolution.

Here we have taken an integrated modeling approach to study conditions for and consequences of fixation of WGD in populations that adapt to an environmental change. Within our Virtual Cell model, we tracked mutations and patterns of genome conservation along the line of descent. WGD was modeled as an ongoing mutation, alongside small scale duplications, deletions and rearrangements, as well as point mutations. Lineages arising from identical ancestral populations alternatively evolved with and without WGD, allowing for a direct comparison of the two modes of evolution.

Our results show that fixation of a WGD increases the likelihood that a population will readapt successfully to a novel environmental condition. Surprisingly, the ancestral gene content of WGD lineages declines more slowly than that of lineages without WGD, while per gene mutation rates were higher in WGD lineages. At the same time, we found that ohnologs were over-retained relative to expectations based on random losses. This effect was strongest for TFs. In agreement with predictions from the gene balance hypothesis we found that TFs with many outgoing interactions were most likely to remain in duplicate. Because very little subfunctionalization was detected in these TFs we concluded that selection for dosage balance caused the over-retention pattern. Hence, a relatively simple, biologically inspired model can explain the association between WGD and environmental change as well as the overarching pattern of biased gene retention that is found in an expanding body of phylogenetic studies of paleopolyploidy.

## Results

The Virtual Cell model consists of populations of cells that have structured genomes encoding three basic classes of genes: TFs, metabolic enzymes and pumps. Together, they constitute a simple metabolism that can be regulated by sensory feedback. The metabolism revolves around two simple molecules: a resource (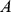) that is available extracellularly and can be transported into the cell, and an energy molecule (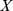) that is produced from the resource and consumed in active transport of the resource into the cell or in an anabolic reaction that channels resource into downstream metabolism. The fitness of cells depends on their ability to keep internal 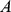 and 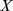 concentrations at a constant target level while environmental 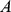 concentrations fluctuate. The performance of a cell in a particular resource condition depends on minimizing the deviation at steady state in 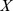 and 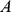 internal concentrations from the targets. A cell's fitness is a function of the performances in up to three environments seen in the lifetime of a cell (explained in more detail in Materials and Methods). To do well under the wide range of fluctuating resource conditions, cells have to evolve tightly regulated resource import and metabolism to maintain their homeostasis.

Mutations occur both at the gene and the genomic level. At the gene level, point mutations affect binding strength and reaction rates of the molecular processes as well as changing gene regulatory interaction structures. TFs regulate genes when their binding motif matches that on a gene's promoter (discrete), while the strength (continuous) and sign of the regulation are coded for by the TF. Interaction structure and the regulatory effect can be independently mutated. At the genomic level, variable stretches of adjacent genes covering up to a quarter of the genome are duplicated or deleted at a rate of 0.024 per generation or reinserted in a new position (0.048) relative to other genes. Finally, the rate of whole genome duplications is approximately a factor 10 lower than SSD, affecting on average 0.3% percent of the population per generation. Note that the introduction of WGD mutations results in a small upward mutational bias for genome size. Nevertheless, the long term evolutionary trend in our simulations was invariably towards smaller genomes. Per gene mutation rates were identical between simulations and kept constant during evolution.

**Figure 1:**
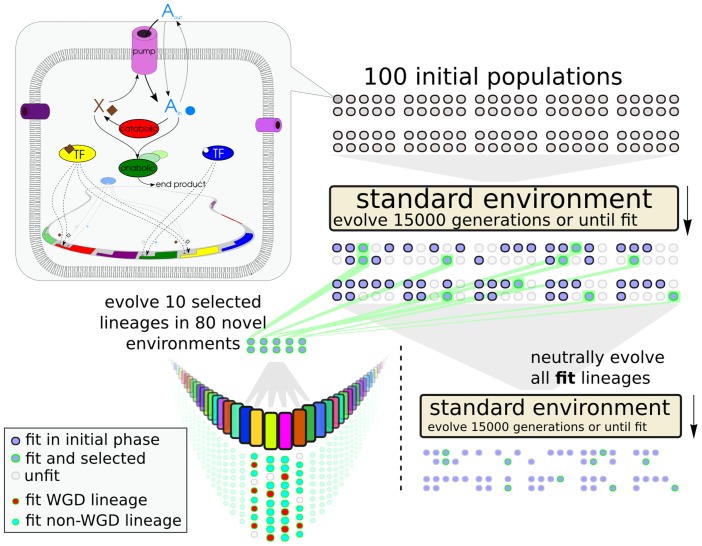
Virtual Cell simulation protocol. A schematic overview of the Virtual Cell model shows how resource 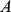 can diffuse and be transported into the cell, where it is converted to energy molecule 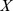 and further catabolized into an unspecified end product. The expression of genes coding for different variants (differently evolved parameters for substrate affinities and binding motifs) of enzymes, pumps and TFs are differentially regulated depending on TF regulatory interactions with their promoters. Genes are located on a spatially explicit, circular genome. For our simulation protocol 100 populations consisting of 1024 cells each were randomly initialized and evolved under standard environmental conditions. From populations that reached high fitness (arbitrarily defined as a fitness higher than 0.85) 10 were selected for further environmental change testing. 1000 generations upon reaching high fitness these 10 selected populations were each subjected to 80 novel environmental conditions. From the 800 simulations fit runs were grouped into lineages with WGD and lineages without WGD within their line of descent. A neutral control set was formed by continuing evolution of the initially fit lineages under identical standard environmental conditions.

In order to study re-adaptation after a change in the environment, we first evolved populations of virtual cells in a standard environment with a fluctuating resource concentration and subsequently let them re-adapt to a large set of novel environmental conditions in parallel evolutionary simulations (Fig. 1). Because the model does not allow for explicit changes in the environment, other than resource fluctuations, we used as a proxy for novel environmental conditions, a set of changes in the non-evolvable parameters of the model. These five parameters that are otherwise fixed are: membrane permeability, protein degradation, energy yield from resource molecules and the two homeostatic targets for 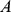 and 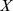. By systematically changing three parameters at a time we created a set of 80 novel conditions that were used to challenge the pre-evolved populations (see Materials and Methods and Table S1 for details about the environments). The fitness criterion was always homeostasis. From a set of 100 simulations performed under standard environmental conditions, we selected 10 populations that evolved highly accurate homeostasis regulation for a broad range of external resource concentrations. Next, for the 10 seed populations environmental change was applied 1000 timesteps after high fitness was obtained. Each was subjected to the 80 novel environmental conditions and allowed to re-adapt. As a control, all fit lineages from the 100 initial simulations, including the 10 populations selected for environmental change testing, also continued neutral evolution, without environmental change, for a further 15000 generations.

### Re-adaptation with WGD

**Figure 2:**
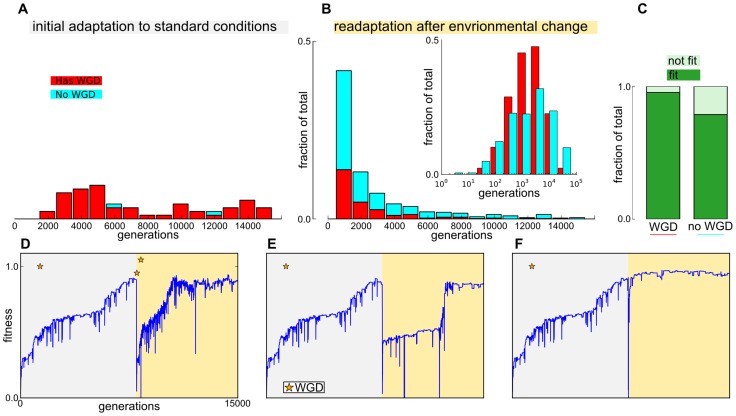
Fitness and whole genome duplication results. (A,B) Simulations binned on the time of reaching high fitness (0.85), when adapting for the first time to standard conditions (A) and re-adapting after an environmental change (B). The bars are split into a fraction that adapted with (red) and without (cyan) a WGD. The inset of B shows the differently shaped distributions of adaptation times on a log-scale for WGD and non-WGD lineages, with WGD lineages showing a sharper peak at intermediate adaptation times. (C) Fractions of runs that became fit after environmental change, separated for runs with and without an ancestral WGD respectively. D,E and F show fitness evolution of three example simulations, where the initial adaption (gray background) is the same and subsequent re-adaptation occurs in different environments (yellow background). We show an example of re-adaptation with additional rounds of WGD at an intermediate time scale (D) and re-adaptation without WGD (E,F) on a longer timescale (E) and on very short timescale of a few tens of generations, after a sharp fitness drop (F).

Initial adaptation times varied widely and those that reached high fitness within 15000 generations almost always involved one or more WGDs (Fig. 2A). In contrast, re-adaptation times for lineages after environmental change were much shorter (Fig. 2B, D–E). More than 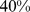 reached high fitness within 1000 generations. This was surprising, because at the start of re-adaptation, fitnessess dropped on average below the level of randomly initialization starting populations. In addition, in successfully re-adapting lineages WGD events became fixed in a minority of lineages, being particularly rare in rapidly re-adapting lineages (Fig. 2B inset, F). The cases with rapid re-adaptations suggest that mutational paths to new phenotypes can be very short, requiring very little change at the genomic level. Notwithstanding the near absence of WGD in rapidly re-adapting lineages, fixation of WGD in the line of descent improved the overall success rate of re-adaptation from 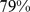 to 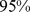 (Fig. 2C). Even though WGD-mutants were generated continuously in the population throughout the evolutionary experiments, very few WGDs were ultimately accepted in the line of descent of the final population. Accepted WGDs occured almost exclusively (in 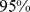 of cases) within 500 generations of the environmental change. The much shorter time scale of genomic expansion relative to the timescale of full re-adaptation is in agreement with our previous work on the Virtual Cell model, showing that early evolution of large genomes generally resulted in better long term evolvability [38].

### Evolution of gene content

**Figure 3:**
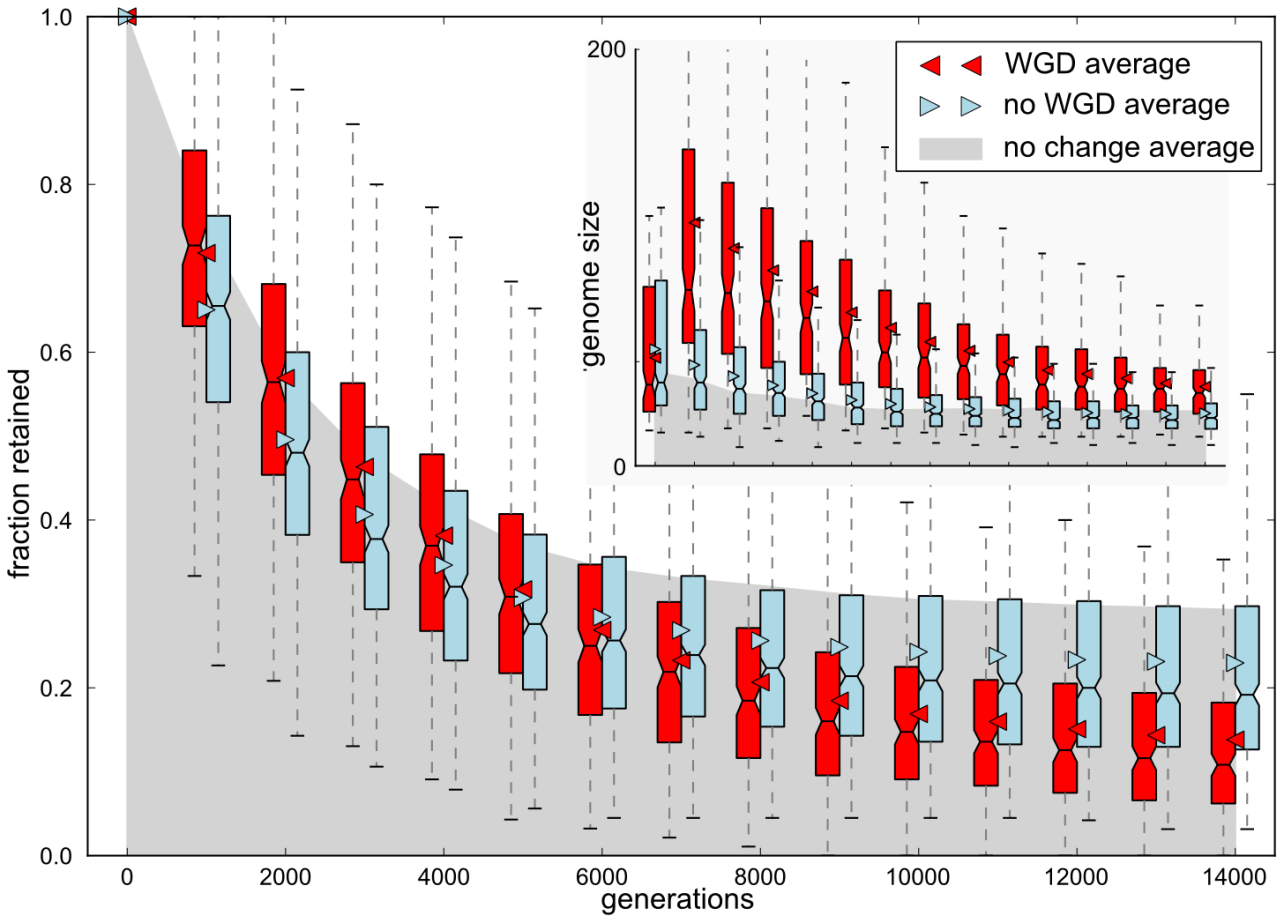
Fraction of conserved ancestral gene content through evolutionary time. The gene content of ancestors at the time of environmental switch was used as reference. At 1000 generation intervals, the overlap in gene content of descendants with the reference was measured. All genes inherited one to one from the ancestral reference (not counting copies from subsequent duplication events) count towards the retained fraction of the total ancestral gene content, in WGD lineages (red), non-WGD lineages (blue) and a neutral control set where the environment was kept the same (gray). Boxes and whiskers show the 50% (box) and 75% (whiskers) ranges of the data around the median (line). Triangles and the upper edge of the shaded area show the averages of the environmental change and neutral evolutionary runs, respectively. The inset shows the distribution of genome sizes.

To study the adaptation process following WGD in more detail we analyzed the evolution of gene content after the environmental change was applied. For all populations the environmental change took place 1000 generations after a fitness 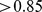 was first recorded in the population. At that time the genome was typically several fold larger than the minimum genome size reached towards the end of the simulation as a result of long term streamlining (Fig. 3 inset). Our previous work on the Virtual Cell model showed that streamlining reduces mutational load by the removal of redundant genes and a focussing cellular function into a small set of essential genes [38], explaining how a relatively large proportion of ancestral gene content is lost during the re-adaptation to the novel environment (Fig. 3).

The conservation of gene content was measured as the fraction of genes in the ancestor, alive during the environmental change, that was maintained in subsequent descendants. Duplicates that arose from WGD and SSD later in evolution were not included in counts of ancestral gene content. As expected, continued neutral evolution in the control set led to drastic streamlining and turnover of the genome, resulting in the loss of approximately two thirds of the original gene content (Fig. 3: gray shaded area). Re-adaptation to environmental change led to even larger changes in gene content, as expected. However, in contrast to our expectation that WGD copies are functionally redundant, a larger fraction of ancestral gene content was conserved in WGD lineages than in non-WGD lineages for more than 5000 generations after environmental change (Fig. 3). This was despite the fact that the per gene deletion rate remains constant with differences in genome size (see Methods). Also, on the long run, the average conserved fraction in WGD lineages, although dropping below that of non WGD lineages, always remained above half the conserved fraction in non-WGD lineages. This shows that at least some fraction of the ancestral content was selectively retained in duplicate.

### Effective mutation rates

**Figure 4:**
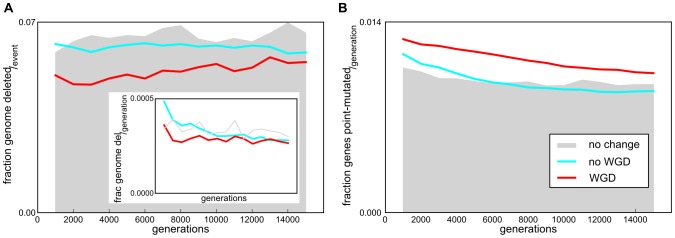
Deletion and point mutation rates in the line of descent. (A) Rate of genes deleted per accepted deletion event as a fraction of total genome size. These fractions were averaged for all fit runs with (red) and without (cyan) a WGD as well as a control set of runs that continued without environmental change (gray), and binned in 1000 generation intervals. In the inset, accepted deletion fractions are per generation instead of per event. (B) Accepted point mutation rates per generation as a fraction of total genome size. The fractions were averaged for all fit runs with (red) and without (cyan) a WGD as well as a control set of runs that continued without environmental change (gray), and binned in 1000 generation intervals.

To find an explanation for the difference in gene content conservation between WGD and non-WGD lineages we analyzed the fixation of different mutation types. The frequency of accepted deletions in neutral, WGD and non-WGD lineages were very similar, although slightly lower for WGD lineages over the whole simulation interval, compared to non WGD and neutrally evolving lineages. However, the fraction deleted per event, for mutations that were accepted, was much smaller for WGD lineages than for neutrally evolving and non-WGD lineages (Fig. 4A; 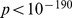) despite this fraction being equal in the background mutations for all three categories. The result was that smaller fractions of the genome were lost per generation in WGD lineages (Fig. 4A inset; 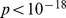).

In contrast, the rate at which point mutations were accepted was significantly higher in WGD lineages compared to non-WGD lineages (Fig. 4B; 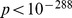), while for the latter, this rate was again very similar to that in the neutral control set (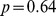). This suggested that individual genes diverged much faster in WGD lineages than non-WGD lineages. In summary, WGD appeared to promote the conservation of duplicated genes, while at the same time enabled genes to diverge more and change their function. A process that fits with these two characteristics is subfunctionalization. To investigate whether the contrast between gene content conservation and higher gene function divergence in WGD lineages could be explained by a subfunctionalization process we focused our subsequent analysis on the fates and divergence of the ohnologs.

### Ohnolog retention

**Figure 5:**
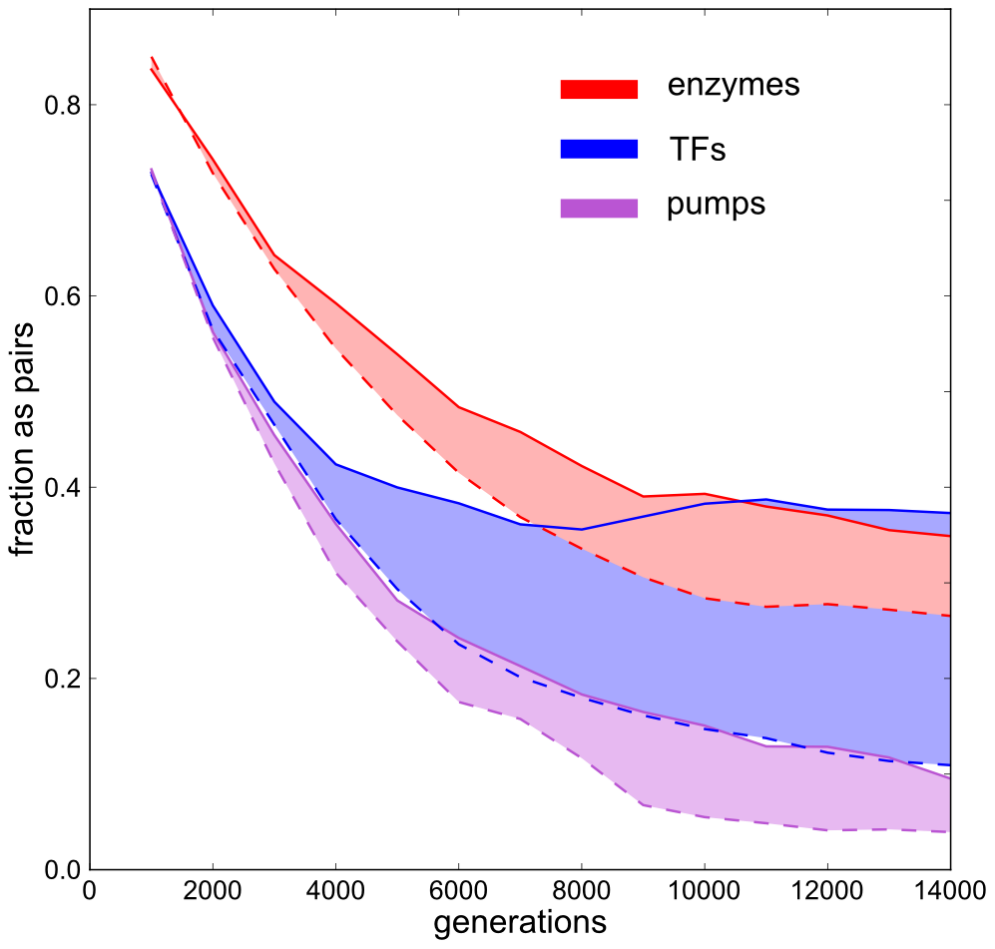
Intact ohnologs as a fraction of conserved WGD content. Of the conserved gene content from the ancestral reference genome the fraction of genes in complete WGD pairs (ohnologs) was plotted, per gene category. Solid lines show the intact ohnologs in the evolutionary data, while dashed lines represent the ohnolog fractions obtained when the same number of deletions per category were applied randomly and in the absence of selection. Shaded areas highlight the difference between evolutionary and neutral simulation results.

We performed random deletion simulation to find the expected pair retention fractions for TFs, enzymes and pumps, separately. For every evolutionary simulation in our test set a random deletion simulation was performed that had the genome configurations of the common ancestor at the time of environmental change in the evolutionary run as its starting point. In the random deletion run, equal amounts of deletions per gene class were performed to those found in the line of descent in the evolutionary run, but selection was omitted. The random deletion runs were pooled in the same way as the evolutionary runs to make comparisons. As shown in Fig. 5 the expected fractions of ohnologs after randomly selecting genes for deletion are much lower than in the evolutionary simulations. Over-retention is highly significant in the case of TFs (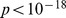) and detectable in enzymes and pumps (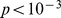; 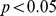). Despite the difference in the strength of the bias between TFs and enzymes, the fraction of these respective gene types that is conserved as ohnologs is very similar toward the end of the simulation (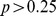), although the fraction has stabilized for TFs, while it is still declining for enzymes. This can be understood by the fact that the rate of deletions is much higher for TFs than for enzymes, resulting in a shift towards higher fractions of enzymes and lower fractions of TFs in the late, streamlined descendants (Fig. S1). Thus, even though TFs were on the whole more likely to be removed from the network by streamlining, the TFs that were conserved at long evolutionary timescales were much more likely to remain in the genome as ohnologs. In the next section we will test the hypothesis that TF connectivity is the determining factor for the retention of TF ohnologs.

### TF connectivity

**Figure 6:**
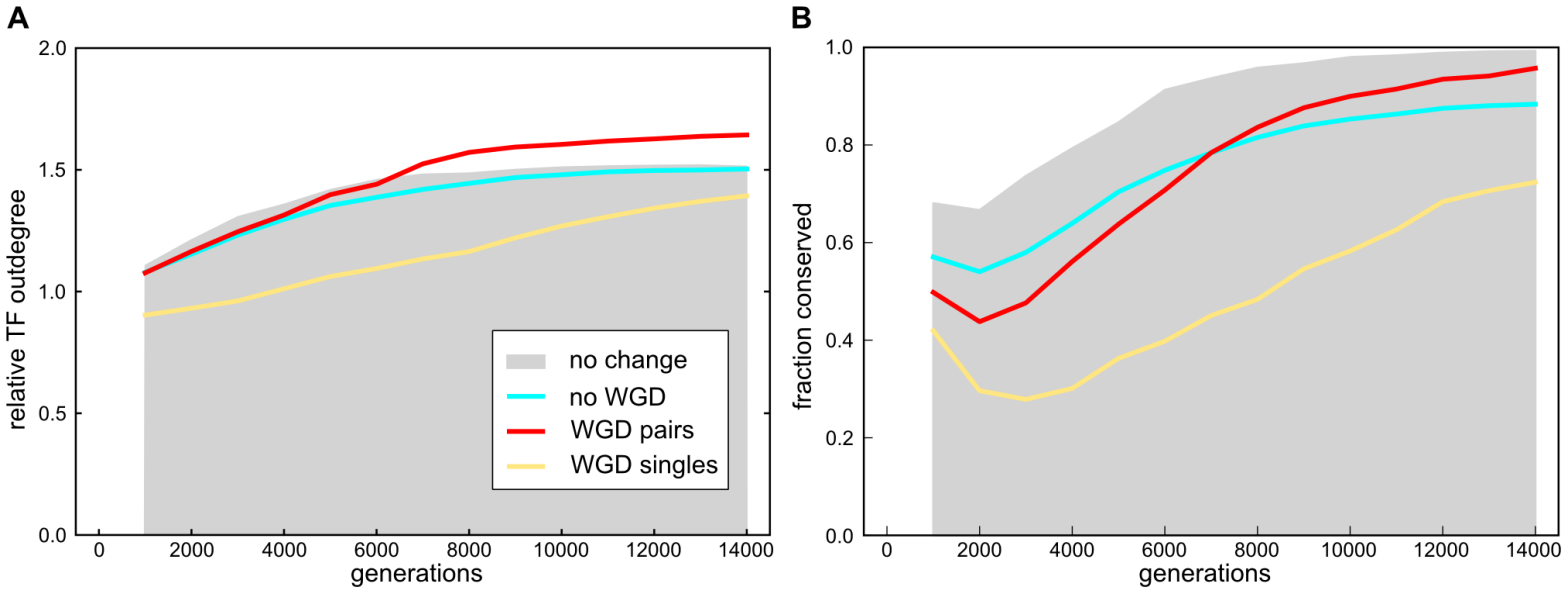
Ancestral outdegree and binding site conservation of conserved TFs. At the time of WGD all TFs were identified and their outdegree and binding site motifs recorded. At 1000 generation intervals conserved TFs from the time of WGD or, if there was no WGD, from the time point when environmental change was applied for the re-adaptation experiments, were identified in the genomes in the line of descent. (A) The average ancestral outdegree of the retained TFs (recorded at the reference time point) was divided by the average outdegree of the all ancestral TFs, thus providing a measure of the influence of ancestral connectivity on the rate of conservation of TFs. For WGD lineages this relative outdegree of conserved TFs was measured separately for ohnologs (red) and singles (yellow). For comparison, the analysis was also done for non-WGD (cyan) lineages and a neutrally evolving control set (gray). (B) The current and ancestral binding sequences of the TFs were compared and conservation score set to 1 if they remained the same and 0 otherwise and all scores averaged per individual.

In the neutral control set continuous streamlining is responsible for the pattern of increasing TF outdegree (Fig. 6A gray shaded). TFs with a relatively high outdegree remained in the genome at the expense of more sparsely connected TFs, which was also true for WGD and non-WGD simulations. Despite going through environmental change, the evolutionary pattern of non-WGD (cyan) lineages is very similar to the neutrally evolving controls. For the WGD lineages, the connectivity of retained genes was calculated separately for ohnologs (red) and singles (yellow), revealing a marked difference in their evolved connectivity (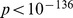). Significantly higher connectivities of ohnologs compared to those of conserved genes in non-WGD (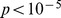) and neutrally evolving lineages (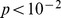) suggests that ancestral connectivity influences duplicate retention post-WGD. At the same time, singles in WGD lineages had significantly lower connectivities, both compared to the ohnologs and the conserved genes in non-WGD (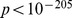) and neutral lineages (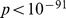).

These results raised the possibility that the observed biased retention of TF ohnologs was a side effect of the conservation of highly interacting genes. To test this, we performed additional random deletion experiments. Now, instead of having an equal probability for each TF to be deleted, deletion probability was made dependent on the ancestral TF connectivity. The probabilities were determined by looking at the distribution of connectivities in the ancestral network and determining the fractions of conserved genes per connectivity bin, at subsequent points in evolutionary time (see Methods for details). Performing such a simulation on the ancestral networks produced connectivity changes over time that were highly comparable to the evolution of connectivity in the evolutionary runs. Importantly, however, adding connectivity bias to the random deletion experiment did not change the result that ohnologs were over-retained in the evolutionary simulations (Fig. S2). We therefore concluded that conservation of highly connected TFs alone could not explain the over-retention of TF ohnologs.

### TF divergence

Continuing our investigation of the role that subfunctionalization may have in the conservation of gene content and high levels of divergence at the gene level in WGD lineages we investigated the functional divergence of TF ohnologs. If both ohnologs would diverge in function at the same time, they would no longer be able to fully compensate for each other's loss, making the conservation of both more likely. Functional divergence of a TF could happen if its binding site (BS) changes and it starts to regulate a different gene set.

In general, BS divergence of ancestral genes was substantial on a short timescale, even in neutrally evolving lineages. Later, however the initial divergence was largely undone (Fig. 6B). This reversal of initial divergence can be attributed to the long term genome streamlining that is expected to remove redundant and non-functional genes [38], expected to be enriched in highly diverged genes. Compared to lineages that did not undergo an environmental change, BSs of re-adapting lineages initially diverged much more initially, highlighting the fast pace of evolutionary change immediately after the environmental change (Fig. 6B). Interestingly, WGD lineages had significantly higher levels of BS divergence compared to non-WGD lineages on the shorter timescale, both in the ohnologs (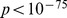) and singles category (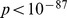). Subsequently, the remaining ohnologs showed a drastic reduction of the level of divergence, eventually reaching a BS conservation level above that of conserved genes of non-WGD lineages (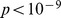). The sharp reduction in average BS divergence indicated that fast diverging ohnologs were overwhelmingly lost, while ohnologs that, on the other hand, did not mutate away from their ancestral BS were conserved at long evolutionary timescales. When one of an ohnolog pair is deleted in the course of evolution the remaining gene is subsequently categorized as a single. It is therefore not surprising that the singles category had a final level of BS divergence that was much higher than that of ohnologs (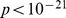), receiving an influx of highly diverged genes from the ohnolog category. However, their long term conservation may in fact be best explained by their diverged role in the network.

It suggests an interesting dual character for the dynamics of post WGD genome evolution. On the one hand, selection acted to conserve highly interacting genes most strongly, both in their copy number and their interaction partners. At the same time, genes of lower connectivity may be returned to a single copy status and diverge in their role within the gene network. The latter process may be particularly important for adaptation in a new environment. Together, our results show that the conservation duplicate pairs and interaction partners of highly connected genes is compatible with the gene balance hypothesis, while subfunctionalization did not play a significant role in pair retention in our model. Instead, most functional divergence was observed in genes conserved as singles after WGD. Apparently these were more free to evolve and adapt to the new environment than ‘singles’ in non-WGD lineages, in congruence with the overall higher rates of divergence in WGD lineages compared to non-WGD lineages (Fig. 4).

## Discussion

In this study we have taken an open-ended approach to studying the relationship between drastic changes in the environment and the occurrence of WGD in the line of descent. Evolving lineages could potentially follow many different evolutionary paths to re-adaption as a result of mutations at multiple scales and a complex genotype to phenotype map. WGD, despite being an ongoing mutation, was observed exclusively in lineages that were still ill-adapted to the prevailing environment, as was the case early during the initial adaptation phase and shortly after an environmental change (e.g. Fig. 2D). This mirrors phylogenetic studies linking WGD to environmental and other types of drastic intracellular change [16], [15], [20], [19], [39].

One or more WGDs occurred during the initial adaptation phase in almost all lineages that would eventually obtain high fitness. In contrast, a minority of lineages (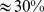) fixed a WGD following environmental change, while almost no WGDs were observed when re-adaptation was very rapid. This indicates that some of the imposed environmental changes were more easily met by a relatively minor recalibration of the pre-evolved regulatory circuits, despite causing severe initial drops in fitness. Nevertheless, successful re-adaptation was more prevalent in lineages with WGD, and consequently larger genomes. This corroborates our previous research, showing that large genome increases early during adaptation benefit long term adaptation [38] and is in accordance with a similar inference drawn by Van de Peer and co-workers [21], [20] based on parallel paleopolyploidy events in plants and frequent species radiation in the wake of WGD [40], [33], [41], [5]. A particular case in point of long term evolvability due to WGD is the evolution of novel signaling and developmental pathways in vertebrates [42], [43], [24].

In addition to the long term benefits, in most lineages immediate positive fitness effects also played a role in establishing WGD (Fig. S3). Moreover, WGD was more frequent after particular types of environmental change, most notably when enzyme degradation rates increased (Fig. S4). This again parallels observations from the phylogenetic record and experiments. For example, there is strong evidence that the ancient WGD in yeast had an immediate benefit in the context of newly evolved fruiting plants [18], [19]. Moreover, short term fitness advantages appear to play a role in establishing polyploid lineages in founder populations within newly arisen environments [16], [15], [44], [17].

Strong genome streamlining occurred in all simulations, irrespective of environmental change and the fixation of WGD, indicating that maintenance of large genomes comes at a considerable mutational cost [44], [38], [45]. However, WGDs create “irremediable complexity” [46], [28], [47], enforcing the maintenance of larger genomes, which would put lineages that evolve to equal fitness without WGD at an advantage. This may explain the relatively low fraction of WGD lineages in our experiments and could be the reason that, although polyploids are widespread among current plant species, their long term survival rate tends to be lower than that of non-polyploids [14].

Summarizing, our simple Virtual Cell model shows a pattern of occurrence of WGD very similar to that in the expanding record of established WGD events in extant organisms. We conclude that it is a generic property of the evolutionary process irrespective of particular evolutionary contingencies and most biochemical constraints. Our results highlight the intricate interplay of short and long term adaptive evolution as well as neutrality and irremediable complexity in shaping the gene content. This is moreover apparent from the duplicate retention pattern, as discussed below.

The fractions of ancestral genes that remain in WGD pairs were higher in all functional categories compared to a neutral expectation based on random deletions. Duplicate retention post WGD was strongly biased towards highly interacting genes, a pattern that has been reported for many paleopolyploid species [30], [48], [23], [28], [43], [26], [25]. However over-retention of pairs, in particular in TFs, was much higher than expected from a biased retention of highly connected genes. The maintenance of duplicate pairs therefore needs another explanation and suggests a form of irremediable complexity. The two main explanations being subfunctionalization and, as recognized more recently, dosage balance selection.

We found no evidence that subfunctionalization played a role in WGD pair retention withing our model, as the duplicates remained very similar. This is in contrast to what has been reported in various cases of duplicate retention [29], [30], [31], [49], [50] and the hypothesis that it was the main cause of genome complexification in eukaryotes [51]. There is in fact ample evidence that sub- and neofunctionalization play an important role in cementing the retained duplicates in the genomes of real organisms [30], [27] and promote innovation [43], [24], [52], although evidence exists that competitive interference between the paralogs may impose a significant obstacle to neutral loss of subfunctions [53]. The lack of subfunctionalization in our model can be explained as follows. Subfunctionalization of regulatory interactions would require that TFs can conserve binding interactions with a subset of ancestral sites, while at the same time losing some other sites. As such fine grained alterations of binding motifs was not possible within the current model due to the discreteness of the binding motifs and hence regulatory interactions, it presented a hard case scenario for subfunctionalization. The fact that we still observed over-retention, most prominently in TFs, again suggests the relevance of the dosage mediated retention mechanism. Another indication that dosage effects were important in the evolutionary dynamics was the observation that high protein degradation rates triggered fixation of adaptive WGD.

Dosage balance selection was proposed to account for the inverse relationship between retention of duplicates post WGD and post SSD [37], [34], [28], [54], [55], [26]. Originally, dosage balance selection is expected to affect proteins that are part of larger protein complexes. For complex assembly it is assumed that the relative dosage of the constituents is required to stay within narrow bounds, to prevent the accumulation of incomplete complexes [32], [28], [37]. Therefore single deletions of a duplicate will mostly not be tolerated after WGD, preventing the return to single copy of subunits of large complexes. Interestingly, our results show that resistance to the deletion of a member of a WGD pair was high, even in the absence of protein complex assembly or physical protein interactions, but that it was still a function of the number of its interactions. This indicates that dosage balance drove the retention.

Although weaker than TFs, enzymes pairs were also significantly over-retained post WGD in our simulations. Biased retention of enzyme duplicates has also been reported for the latest of P. tetraurelia's three successive WGDs [23]. Curiously however, enzymes were significantly under-retained from the earlier WGD events. Initially, stoichiometric constraints likely impose dosage balance selection on enzymes in metabolic pathways [56]. However, over longer evolutionary timescales, the enzymatic pathways may acquire compensating expression level changes that free the enzyme duplicates of dosage balance constraints, allowing them to be deleted. Indeed, looking at the trend within the fraction of enzymes found in pairs in our simulations (Fig. 5), the decline phase is longer than for TFs and may have continued with longer simulation times, explaining the varying levels of retention at different evolutionary timescales.

Summarizing, in our simulations dosage sometimes played an important role in establishing adaptive WGD as well as driving the retention of duplicate pairs, conserving core regulatory interactions in the absence of subfunctionalization. This raises the question how novel functions could evolve within our simulations, without significant divergence of conserved ohnologs. The answer appears to be provided by the behavior of the singles in WGD lineages. They were changing much faster than duplicates and also notably faster than genes retained in non-WGD lineages (Fig. 6B). This opens the possibility that the adaptive success of WGD lineages was in part due to more sparsely connected TFs (Fig. 6A) that were not essential for fitness and were therefore more free to evolve. These are expected to be in relative abundance immediately after a WGD. This scenario can, moreover, explain the result that even though genome conservation was higher in WGD lineages, individual genes appeared to diverge faster from the ancestral state. Thus, enhanced evolvability of WGD lineages was not primarily a consequence of ‘freeing’ redundant paralogs to adopt new functions, but most importantly due to unhindered evolution of non-paralogous genes to adapt to novel conditions.

An important aspect of polyploidization that was left out of our modeling is the variety of mechanisms that can lead to polyploidization. WGD in the current model happened exclusively through autopolyploidization, causing a strict duplication of the genetic material. In contrast, hybridization between individuals from substantially diverged subpopulations can give rise to important phenomena such as biased fractionation patterns [57], [58] and hybrid fitness [59], [60]. We envision that incorporating these mechanisms into the current model could give insight into the adaptive consequences of hybridization events and help recognize the type of ancient polyploidization events by observing characteristic patterns of genome evolution.

Concluding, our model highlights how the interplay between short and long term adaptive and neutral processes underlies the presence of WGD and post-WGD gene retention and its role in genome complexification. Although we did not set out to model this property explicitly, dosage effects and selection for retaining balanced gene expression readily emerged in the Virtual Cell model, underlining its importance as a generic property of evolution, shaping the content of genomes. In addition, WGD appears to enable the divergence of singly retained ancestral genes, which may endow WGD lineages with long term adaptive benefits. From a broader perspective, our results suggest that WGDs had a defining role in enabling the innovations in eukaryotic lineages, while preserving the hallmarks of their ancestors.

## Materials and Methods

Our Virtual Cell model is an adaptation of the work by Neyfakh *et. al* [61] and previously described in [38]. The source code of the model is available at https://bitbucket.org/thocu/virtual-cell. Virtual cell internal dynamics are governed by five basic protein types and two molecular types, 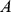 and 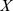. The resource (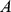) that is present in the environment can be a source of energy when it is enzymatically converted into the energy carrier molecule 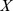. Alternatively 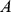 and 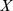 can converted to building blocks in a second enzymatic reaction. The resource diffuses passively over the cell membrane, but can also be actively pumped inwards by pumps consuming 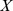. Two types of transcription factors are distinguished by their ligand, 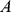 or 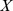, respectively. TFs bind to a gene regulatory region depending on a match between the binding sequence of the TF and the operator region of the gene. Regulatory effects can be positive or negative and the effect differs between the ligand bound and ligand free state. All enzymatic reactions are modeled by ordinary differential equations (see Supplementary Text). Ligand to TF and TF to operator binding are assumed to be fast processes that are in quasi steady state.

Cell fitness depends on their ability to maintain homeostasis in 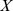 and 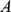. Because cells experience fluctuations in resource concentrations, they need to evolve regulatory circuitry feeding back on the expression of metabolic enzymes and pumps. Cell fitness is measured as follows. Deviation from the target at steady state in a particular environment is calculated as: 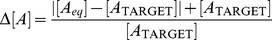 (and analogously for 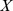) and the measure of fitness in each resource condition 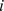 experienced by a cell during its life is inversely proportional to this deviation: 
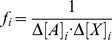
. Fitness of a cell is the non-decreasing function 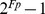, where 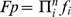 given the set of resource conditions 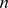 it has seen. Every generation all cells reproduce with a chance proportional to their fitness, until the offspring completely replaces the previous population.

Genotypes are subjected to three distinct types of mutations. The first type alters the parameters of individual genes and is comparable to a point mutation. Affected parameters are the rate and binding constants of enzymes and binding sequences of TFs and promoter regions as well as the ligand that TFs have. The second type of mutation are duplications, deletions and excision insertion that affect stretches of the genome of up to 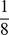. Finally, WGD affects on average 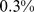 of the population per generation. Finally, point mutations affect genes at a rate 5 times higher than large scale events.

### Environmental change simulations

The evolutionary simulations were run in two stages. In the first stage 100 populations were randomly initialized and independently evolved until 1000 generations after they passed the high fitness cutoff, continuing to a maximum of 15000 generations. All populations in this batch were evolved under the same standard environmental conditions (the same as those in [38]). From the populations that evolved to a high fitness 10 were randomly selected to go to the next stage. In this stage all ten populations were initially cloned 80 times and each cloned populations uniquely assigned to 1 out of 80 novel environmental conditions, per seed population. After applying the environmental change, populations evolved a further 15000 generations. As a control for the effect of environmental change, evolution was continued without environmental change for all populations from the batch of 100 simulations that evolved to high fitness, including the 10 selected seed populations.

### Environmental change set

The new environments were made by changing the values of five parameters of the system relative to their standard values. These parameters separately control the degradation rate of enzymes, permeability of the resource molecule (A), the internal target concentrations for homeostasis in X and A and the metabolic conversion rate of A to X. For all 5 parameters low and high conditions were chosen by making them a factor 2 to 4 different from the standard environment, depending on the severity of the effect on populations in test simulations (see Table S1). For some parameters, too large changes resulted in non-viable conditions for most populations, constraining the change we could effectively apply in our simulations. Finally, a systematic set of 80 environmental changes was constructed by making all 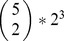 combinations where exactly three parameters differ from their value in the standard condition.

### Ancestor tracing and effective mutation rates

Exact counts of mutations can be traced in the line of descent. To do this, one individual of the final population is selected and its ancestors traced back to the start of the simulation. Mutation events in the line of descent are converted to rates by averaging mutations over 1000 generation intervals.

### Ancestral gene content conservation

The ancestral gene content conservation was measured using the ancestor trace. The gene content of the ancestor that was alive when the environment was changed is the point of reference. The genomes of individuals in the line of descent was overlapped with the reference and what remains of the ancestral gene content was expressed as a fraction. In the case of WGD or other types of duplication of ancestral genes, only one (random) copy is considered the original, while the other copy does not count towards the ancestral content.

### Ohnolog retention and background expectation

After WGD the genes that originated at the WGD event were traced in the line of descent. Again, duplicates that arose subsequent to this reference point were not counted towards ancestral WGD genes. The WGD genes that had remained in the genome were then divided into singles and ohnologs. Duplicate retention fractions are the intact ohnologs divided by the total amount of ancestral WGD genes still present in the genome.

The background expectations were calculated by counting the losses per gene category of ancestral WGD genes along the line of descent and then resimulating the losses, starting from the first ancestor with the WGD. In the first variant of the random deletion simulations, the deletion counts were performed entirely randomly per gene category on the ancestral content. In the second variant, the probabilities of selecting a gene for deletion were made dependent on their connectivity. The scaling was calculated directly from the original evolutionary experiments, by assigning genes (TFs) to bins according to their relative connectivity in the ancestral WGD network and finding the likelihood of deletion in each connectivity bin. This resulted in comparable connectivity curves for the connectivity adjusted random deletion experiments and the evolutionary simulations.

### Significance testing

Significance of reported differences between WGD, non-WGD and neutrally evolving lineages were all determined by Mann-Whitney rank sum tests, ranking the scores of individual runs within each of the subsets. In the case of mutation rates, the ranking was done over the full evolutionary time interval, while in the case of ohnolog conservation, TF connectivity, and BS divergence ranking was performed on the scores of the last time point, unless stated otherwise in the main text.

## Supporting Information

Figure S1 **Average fractions and absolute numbers of TFs, enzymes and pumps in non-WGD, neutrally evolving and WGD lineages.** In all three types of population the trend is towards larger fractions of enzymes in the genome, at the expense of TFs. This occurs irrespective of large differences in the maximum genome sizes in different types of populations.
(TIFF)

Figure S2 **TF ohnologs as a fraction of total conserved WGD gene content.** Results from two types of neutral deletion simulations are compared with the evolutionary data. In the first deletion simulation (dashed line), the rate of deletions is equal to that in the evolutionary data, but TFs are deleted randomly. In the second type of simulation, the probability of deleting a TF depends on the outdegree of the TF within the reference genome of the WGD ancestor (see also the Materials and Methods section).
(TIFF)

Figure S3 **Fitness effects of WGDs in the line of descent.** For all WGD events that were accepted in the lines of descent of the complete set of simulations the change in standard fitness was recorded. Because resource concentrations vary stochastically during the life time of a cell, the actual fitness effect experienced by the cell was different from the standardized effect (see Supplementary Text section: Fitness evaluation and reproduction).
(TIFF)

Figure S4 **Fitness and adaptation speed after different types of environmental change.** Data points represent averaged fitness (x-axis) and WGD count (y-axis) of the ten seed populations in each of the 80 environments. On the z-axis are the three levels of degradation rate used in the environment set. We categorize environments as fast adapting, when at least 8 out of 10 seed populations readapt to the environment within 1000 generations. The different marker styles indicate the relative scaling of the *A* and *X* homeostasis target values, where blue diamonds indicate that the targets are at equal height, as is the case in the standard environment, red triangles that there is a 4 fold difference and green circles indicate a 16 fold difference in target values. Several patterns can be observed. First, the low degradation rate seems to provide a hard case for adaptation, having very few fast adapting environments and being associated with low fitness values, whereas the high degradation rate has relatively many fast adaptation points and high fitness values. Secondly, the high degradation rate environment coincides with more WGDs. Thirdly, the environments with a 16-fold difference between the *A* and *X* targets are not in the fast adaptation set and skewed towards lower fitness values. Only when degradation rate is simultaneously high are average fitness values high and did at least some populations have a WGD, within the particular environment.
(TIFF)

Figure S5 **Fraction of ancestral gene content within present genome.** A reference gene content is saved at the time of WGD. At subsequent 1000 generation intervals the genomes in the ancestor trace (see Materials and Methods) are searched for conserved reference genes, without counting duplicates that have arisen after the reference point. The conserved ancestral content is expressed as a fraction of the complete gene content at every time point.
(TIFF)

Table S1 **Parameter values used in standard and changed environmental conditions.** For all parameters a high and a low value are defined in relation to the value used in the initial fase of the evolutionary simulations (standard). Environmental change conditions are generated by sampling from the three parameter levels. The construction of the full environmental change set used for simulations is described in the Materials and Methods section.
(PDF)

Text S1 **Detailed description of the evolutionary model and ODE system for internal cellular dynamics.**
(PDF)
