# Supplementary figures and images for: A Synergism between Adaptive Effects and Evolvability Drives Whole Genome Duplication to Fixation
Source: PLoS Comput Biol. 2014 Apr 17;10(4):e1003547. doi: 10.1371/journal.pcbi.1003547 (PMC3990473; doi:10.1371/journal.pcbi.1003547)

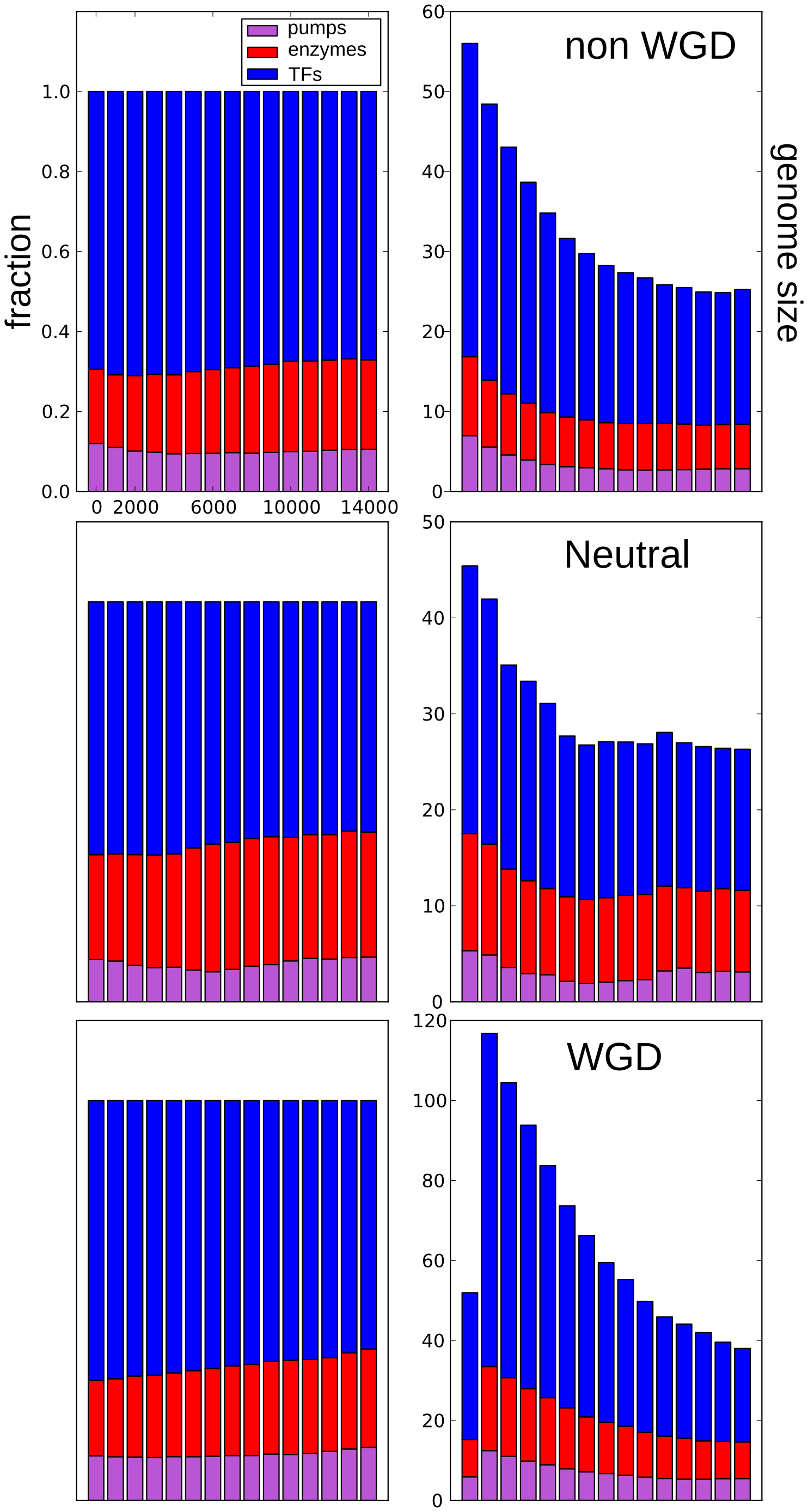

Supplement: Figure S1 — Average fractions and absolute numbers of TFs, enzymes and pumps in non-WGD, neutrally evolving and WGD lineages. In all three types of population the trend is towards larger fractions of enzymes in the genome, at the expense of TFs. This occurs irrespective of large differences in the maximum genome sizes in different types of populations. (TIFF) [file pcbi.1003547.s001.tiff]

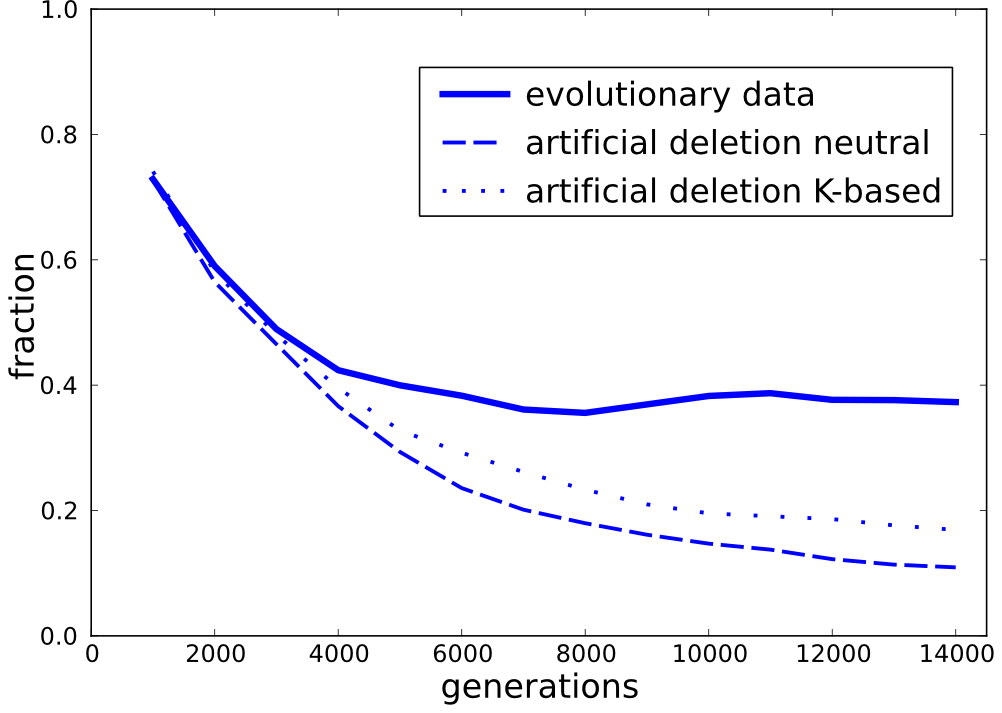

Supplement: Figure S2 — TF ohnologs as a fraction of total conserved WGD gene content. Results from two types of neutral deletion simulations are compared with the evolutionary data. In the first deletion simulation (dashed line), the rate of deletions is equal to that in the evolutionary data, but TFs are deleted randomly. In the second type of simulation, the probability of deleting a TF depends on the outdegree of the TF within the reference genome of the WGD ancestor (see also the Materials and Methods section). (TIFF) [file pcbi.1003547.s002.tiff]

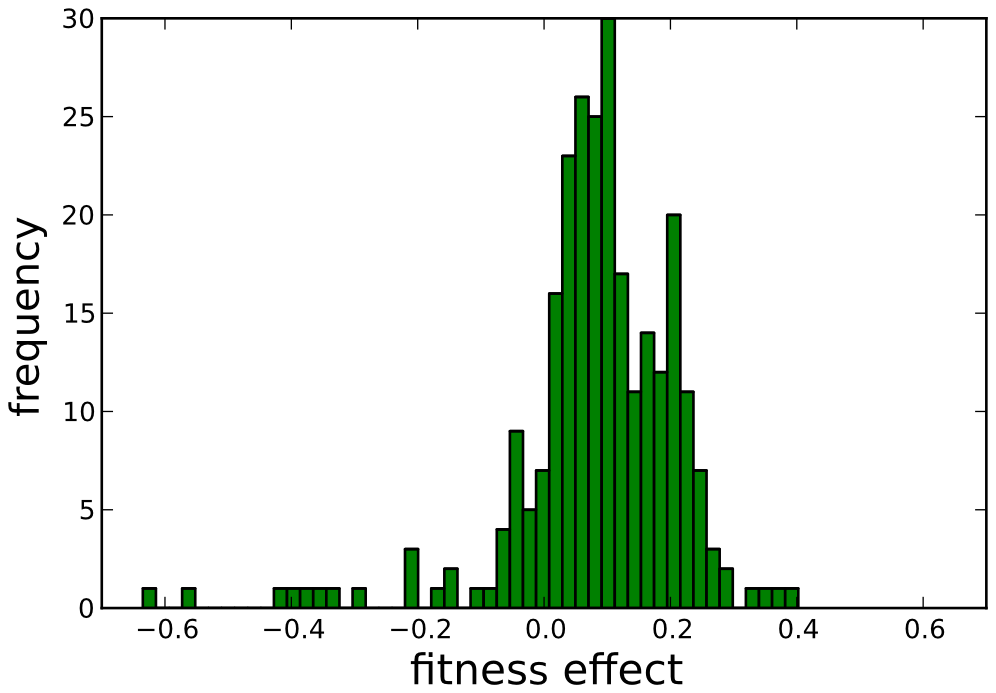

Supplement: Figure S3 — Fitness effects of WGDs in the line of descent. For all WGD events that were accepted in the lines of descent of the complete set of simulations the change in standard fitness was recorded. Because resource concentrations vary stochastically during the life time of a cell, the actual fitness effect experienced by the cell was different from the standardized effect (see Supplementary Text section: Fitness evaluation and reproduction). (TIFF) [file pcbi.1003547.s003.tiff]

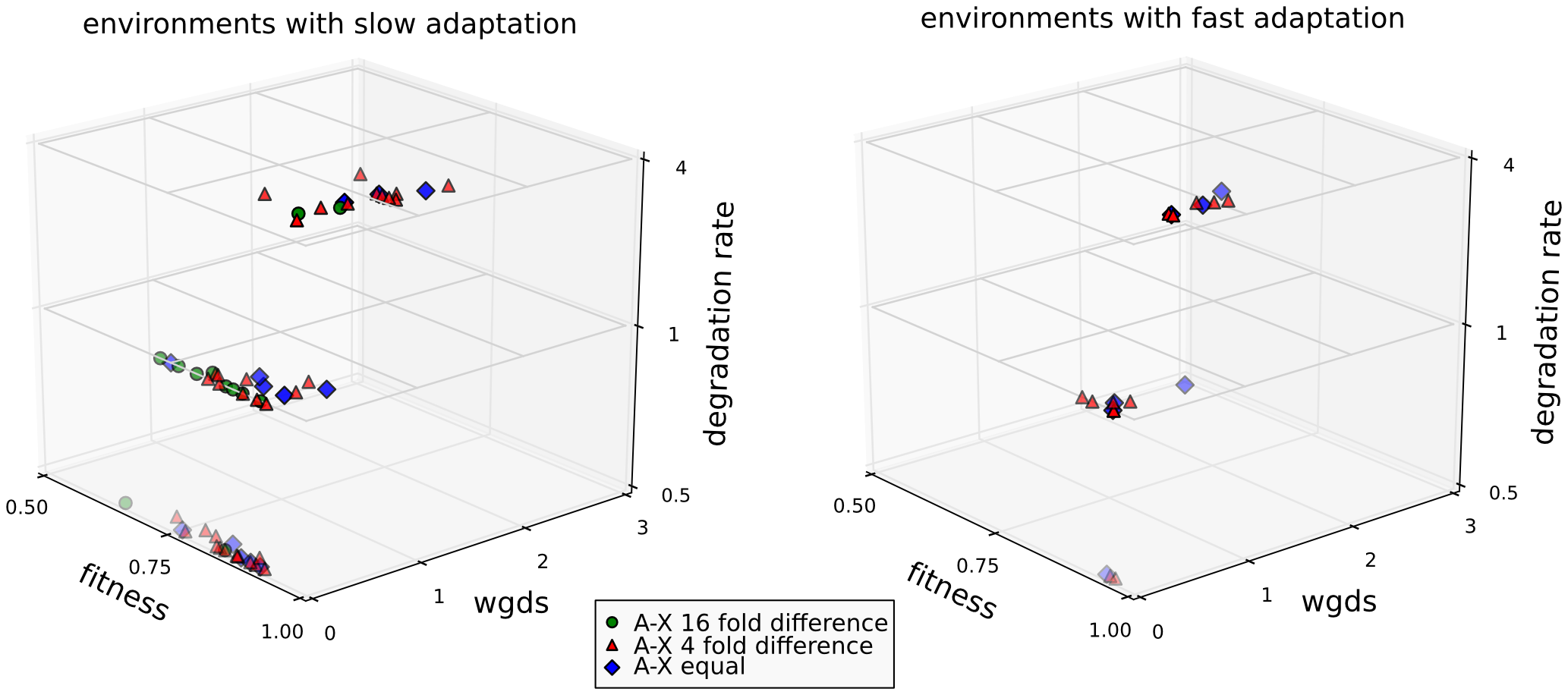

Supplement: Figure S4 — Fitness and adaptation speed after different types of environmental change. Data points represent averaged fitness (x-axis) and WGD count (y-axis) of the ten seed populations in each of the 80 environments. On the z-axis are the three levels of degradation rate used in the environment set. We categorize environments as fast adapting, when at least 8 out of 10 seed populations readapt to the environment within 1000 generations. The different marker styles indicate the relative scaling of the A and X homeostasis target values, where blue diamonds indicate that the targets are at equal height, as is the case in the standard environment, red triangles that there is a 4 fold difference and green circles indicate a 16 fold difference in target values. Several patterns can be observed. First, the low degradation rate seems to provide a hard case for adaptation, having very few fast adapting environments and being associated with low fitness values, whereas the high degradation rate has relatively many fast adaptation points and high fitness values. Secondly, the high degradation rate environment coincides with more WGDs. Thirdly, the environments with a 16-fold difference between the A and X targets are not in the fast adaptation set and skewed towards lower fitness values. Only when degradation rate is simultaneously high are average fitness values high and did at least some populations have a WGD, within the particular environment. (TIFF) [file pcbi.1003547.s004.tiff]

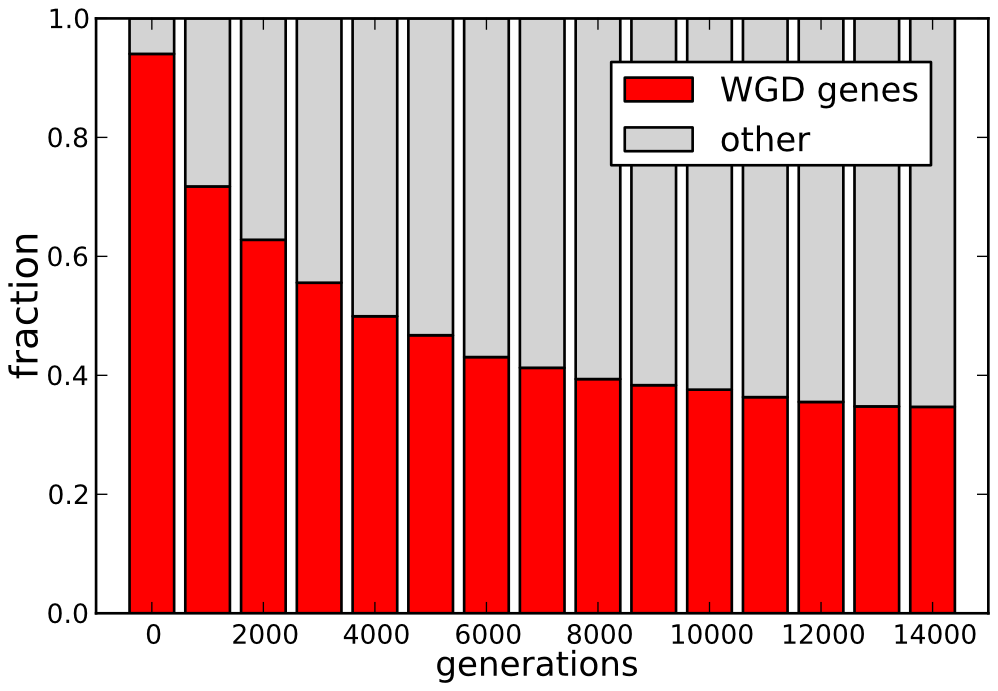

Supplement: Figure S5 — Fraction of ancestral gene content within present genome. A reference gene content is saved at the time of WGD. At subsequent 1000 generation intervals the genomes in the ancestor trace (see Materials and Methods) are searched for conserved reference genes, without counting duplicates that have arisen after the reference point. The conserved ancestral content is expressed as a fraction of the complete gene content at every time point. (TIFF) [file pcbi.1003547.s005.tiff]
