# Supplementary material for: A Synergism between Adaptive Effects and Evolvability Drives Whole Genome Duplication to Fixation
Source: PLoS Comput Biol. 2014 Apr 17;10(4):e1003547. doi: 10.1371/journal.pcbi.1003547 (PMC3990473; doi:10.1371/journal.pcbi.1003547)
Supplement: Table S1 — Parameter values used in standard and changed environmental conditions. For all parameters a high and a low value are defined in relation to the value used in the initial fase of the evolutionary simulations (standard). Environmental change conditions are generated by sampling from the three parameter levels. The construction of the full environmental change set used for simulations is described in the Materials and Methods section. (PDF) [file pcbi.1003547.s006.pdf]

	parameters				
level	permeability	degradation	conversion	X-target	A-target
standard	0.1	1	4	1	1
high	0.4	4	8	4	4
low	0.05	0.5	2	0.25	0.25

Table 1: **Parameter values used in standard and changed environmental conditions.** The construction of the environmental change set is described in the Methods section.
