# Supplementary material for: A Synergism between Adaptive Effects and Evolvability Drives Whole Genome Duplication to Fixation
Source: PLoS Comput Biol. 2014 Apr 17;10(4):e1003547. doi: 10.1371/journal.pcbi.1003547 (PMC3990473; doi:10.1371/journal.pcbi.1003547)
Supplement: Text S1 — Detailed description of the evolutionary model and ODE system for internal cellular dynamics. (PDF) [file pcbi.1003547.s007.pdf]

Virtual Cell model description

Cellular dynamics

The metabolism of the virtual cells revolves around the resource molecule A and energy carrier X. Five protein types govern the intracellular processes of pumping, metabolism and transcription regulation, with each individual gene defining its own set of variables:

Pump enables the uptake of A from the environment by using the energy stored in X. Genes encoding pumps define the following binding and rate parameters:

- $\mathbf{Ka}_{\mathrm{p}}$ binding constant for A_{out}: inverse of $[A_{\mathrm{out}}]$ where half of the pumps are bound by A
- $\mathbf{Kx}_{\mathrm{p}}$ binding constant for X: inverse of $[X]$ where half of the pumps are bound by X
- $\mathbf{Vmax}_{\mathrm{p}}$ rate constant determining maximum influx of A through the pump.

Catabolic enzyme converts resource A into energy carrier X.

- $\mathbf{Ka}_{\mathrm{cata}}$ analogous to Ka_{p}
- $\mathbf{Vmax}_{\mathrm{cata}}$ determines maximum flux through the enzyme

Anabolic enzyme synthesizes an unspecified building block, consuming A and X.

- $\mathbf{Ka}_{\mathrm{ana}}$ analogous to Ka_{p}
- $\mathbf{Kx}_{\mathrm{ana}}$ analogous to Kx_{p}
- $\mathbf{Vmax}_{\mathrm{ana}}$ determines maximum flux through the enzyme

TF two types exist that have A or X as their ligand respectively. A TF regulates the expression of a set of downstream genes.

- **b** A binding sequence type that determines binding to downstream genes.
- $\mathbf{K}_{\mathrm{d}}$ constant of dissociation, inverse concentration at which half of the TFs ligand is bound to it (see below)
- $\mathbf{K}_{\mathrm{b}}$ binding constant that describes the TFs affinity for the downstream operators that it binds to, inverse [TF] where half of the available binding sites are bound (see below)
- $\mathbf{Eff}_{\mathrm{apo}}$ regulatory effect that the TF has in the ligand-free state
- $\mathbf{Eff}_{\mathrm{bound}}$ regulatory effect that the TF has in the ligand-bound state

Each gene moreover encodes a promoter strength (**Pr**) that determines basal transcription rate and an operator sequence (**o**), represented by an integer value, that determines which TFs can regulate its respective expression.

The following cellular processes are modeled using ordinary differential equations:

diffusion over the membrane

$$\frac{d[A]}{dt} = ([A_{\mathrm{out}}] - [A])Perm \tag{1}$$

pumping

$$\frac{d[X]}{dt} = \frac{-d[A]}{dt} \tag{2}$$

$$\frac{d[A]}{dt} = \frac{[A_{\mathrm{out}}] \cdot [X] \cdot Vmax_p \cdot [Prot_p]}{([A_{\mathrm{out}}] + Ka_p)([X] + Kx_p)} \tag{3}$$

catabolism

$$\frac{d[A]}{dt} = \frac{-[Prot_{\mathrm{cata}}] \cdot [A] \cdot Vmax_{\mathrm{cata}}}{[A] + Ka_{cata}} \tag{4}$$

$$\frac{d[X]}{dt} = -N\frac{d[A]}{dt} \tag{5}$$

anabolism

$$\frac{d[A]}{dt} = \frac{-[Prot_{\mathrm{ana}}] \cdot [A] \cdot [X] \cdot Vmax_{\mathrm{ana}}}{([A] + Ka_{ana})([X] + Kx_{ana})} \tag{6}$$

$$\frac{d[X]}{dt} = \frac{d[A]}{dt} \tag{7}$$

protein expression and degradation

$$\frac{d[Prot]}{dt} = Pr \cdot Reg - Degr \cdot [Prot] \tag{8}$$

N determines the yield in X of one molecule of A. In our default simulations it is set to 4. All proteins are degraded with the same fixed rate **Degr** = 0.1. Regulation (**Reg**) of gene expression is a function of all the TFs that can bind that genes operator sequence and calculated as follows:

$$W_{tf_{bound}} = \frac{[ligand] \cdot K_d}{1 + [ligand] \cdot K_d} \tag{9}$$

$$W_{tf_{apo}} = 1 - W_{tf_{bound}} \tag{10}$$

$$V_{o_{tf}} = \frac{[W]_{tf} \cdot K_b}{1 + \sum_{\sigma}^{states} \sum_{i}^{n_o} [W]_{i_\sigma} \cdot K_{b_{i_\sigma}}} \tag{11}$$

$$Reg_{g_o} = \sum_{i}^{n_o} V_{o_i} \cdot Eff_{o_i} + (1 - \sum_{i}^{n_o} V_{o_i}) \cdot 1. \tag{12}$$

Here, W gives the fraction of TF molecules that is bound to or free from its ligand. V is the fraction of time that an operator is bound by one particular TF out of all possible TFs with a corresponding binding sequence (n_o). *states* are the ligand-bound and ligand-free form of TFs. *Reg* for a particular gene with operator o is the sum of all regulatory effects of upstream TFs in their respective states according to the fraction of time they are bound to this operator + the basal transcription effect (1.) when it is not TF-bound.

All differential equations are solved by simple Euler integration, either until an equilibrium steady state is reached or a maximum number of time steps (default=1000) have passed.

Population initialization

We initialize each run with $32^2 = 1024$ individual cells. Individual genomes are randomly initiated with sizes distributed normally around 10. TFs are twice as abundant as the pumps and enzymes in randomly created cells. All binding parameters are bounded between 0.1 and 10, and initialized as 10^a with a normally distributed between -1 and 1. All randomly initialized operators and binding sequences $\in \{1, 2..10\}$.

Environmental variability

In our simulations cells are essayed in 3 resource conditions every generation. Per resource condition the $[A_\text{out}]$ changes to a new value with a probability of 0.4, making the chance that $[A_\text{out}]$ remains constant during one generation 0.6·0.6 = 0.36. $[A_\text{out}]$ takes on values 10^r with r drawn from a normal distribution over $[-1.5..1.5)$, thus ranging over 3 orders of magnitude.

Fitness evaluation and reproduction

As is described above, between 1 and 3 different resource conditions are encountered per generation, which leads to a sparse evaluation of fitness. Fitness of cells is calculated according to their ability to reach steady state levels of $[A_\text{in}]$ ($[A_\text{eq}]$) and $[X_\text{in}]$ ($[X_\text{eq}]$) that approach predefined target concentrations $[A_\text{TARGET}] = 1.$ and $[X_\text{TARGET}] = 1.$, in the standard environment. When no steady state is reached within a maximum number of time steps, a cell is assigned a fitness of 0. Otherwise the differences relative to the targets are recorded as $\Delta[A] = \frac{|[A_\text{eq}]-[A_\text{TARGET}]|+[A_\text{TARGET}]}{[A_\text{TARGET}]}$ and similarly for $\Delta[X]$. The performance of a cell under a resource condition i is given by $f_i = \frac{1}{\Delta[A]_i \cdot \Delta[X]_i}$. Its fitness potential $Fp = \prod_i^n f_i$ given the set of resource conditions n it has seen. Fitness of a cell, defining its reproductive chances, is the non-decreasing function $2^{Fp} - 1$. Every generation all cells reproduce with a chance proportional to their fitness, until the offspring completely replaces the previous population. A standard fitness was defined by testing cells in 3 resource concentrations: $[A_\text{out}] = 0.1$, 1, 10 and calculating fitness as before.

Mutation

After replication, the new cells are subjected to a round of mutation, applying the different mutational operators in a chance process, according to their relative rates. The genome is subjected to point mutations, affecting individual parameters, as well as major mutations that act on stretches of genes. We define an overall mutation rate per gene and specify the relative ratio at which point mutations, duplications, deletions and rearrangements, take place. In our default settings, where the overall genic mutation rate is set to 0.019 and the fractions are equal for rearrangements, duplications, deletions and point mutations we expect $0.05 \cdot \frac{1}{4}$ point mutations per gene per round of mutation, etc. Point mutations alter the various constants (c) with the function $c_{new} = c_{old}^s$ with s drawn from a normal distribution over $[0.1..10)$. The minimum and maximum values that c can take on, however, are 0.1 and 10. Operators and binding sequences, when mutated, take on a new value $\in \{1, 2..10\}$.

The different large scale mutations occur at most once per generation and affect stretches of up to $\frac{1}{8}$ of the total genome size with an average stretch size of $\frac{1}{16}$ of the genome. The probability of an event is scaled to match the per gene mutation rate.
